# Prognostic value of the HALP index, erythrocyte sedimentation rate, and rheumatoid factor in rheumatoid arthritis: A laboratory-based multivariate analysis

**DOI:** 10.5937/jomb0-61610

**Published:** 2026-03-17

**Authors:** Xiaohua Xu, Xiaohua Huang, Lu Yongfang, Zeng Defei

**Affiliations:** 1 Longyan First Affiliated Hospital of Fujian Medical University, Departments of Clinical Laboratory, Longyan, China; 2 Longyan First Affiliated Hospital of Fujian Medical University, Department of Reproductive Medicine, Longyan, China

**Keywords:** rheumatoid arthritis, HALP index, erythrocyte sedimentation rate, rheumatoid factor, biochemical markers, laboratory medicine, reumatoidni artritis, HALP indeks, brzina sedimentacije eritrocita, reumatoidni faktor, biohemijski markeri, laboratorijska medicina

## Abstract

**Background:**

Biochemical and hematological indices play a critical role in evaluating systemic inflammation and disease activity in rheumatoid arthritis (RA). This study investigated the prognostic value of the hemoglobin-albumin-lymphocyte-platelet (HALP) index, in combination with erythrocyte sedimentation rate (ESR) and rheumatoid factor (RF), for predicting RA disease progression.

**Methods:**

A retrospective analysis was performed on 146 RA patients admitted between December 2022 and December 2023. Laboratory variables included HALP index, ESR, RF, C-reactive protein, anti-CCP antibodies, hemoglobin, albumin, lymphocyte and platelet counts, and serum creatinine. Measurements were obtained using standardized automated hematology and biochemical analyzers. Disease activity and radiographic joint damage scores were also assessed. Statistical approaches included correlation analysis, regression analysis, and receiver operating characteristic (ROC) curve evaluation.

**Results:**

The HALP index, ESR, and RF were significantly associated with RA disease progression. The HALP index demonstrated a strong correlation with disease activity (r=0.602, P&lt;0.001). ROC analysis showed an area under the curve (AUC) of 0.858 for HALP, 0.841 for ESR, and 0.924 for RF. When combined in a multivariate regression model, the predictive performance improved (AUC=0.943).

**Conclusions:**

The HALP index, as a composite biochemical marker, in combination with ESR and RF provides significant prognostic information for RA progression. These findings underscore the importance of laboratory-based indices in refining prognostic assessments and highlight their potential role in personalized disease management.

## Abstract (sr)

**Uvod:**

Biohemijski i hematološki indeksi igraju ključnu ulogu u proceni sistemske upale i aktivnosti bolesti kod reumatoidnog artritisa (RA). Ova studija je istraživala prognostičku vrednost indeksa hemoglobin-albumin-limfocit-trombociti (HALP), u kombinaciji sa brzinom sedimentacije eritrocita (ESR) i reumatoidnim faktorom (RF), za predviđanje progresije RA bolesti.

**Metode:**

Retrospektivna analiza je sprovedena na 146 pacijenata sa reumatoidnim artritisom primljenih između decembra 2022. i decembra 2023. godine. Laboratorijske varijable su uključivale HALP indeks, sedimentaciju eritrocita (SE), RF, C-reaktivni protein, anti-CCP antitela, hemoglobin, albumin, broj limfocita i trombocita i serumski kreatinin. Merenja su dobijena korišćenjem standardizovanih automatizovanih hematoloških i biohemijskih analizatora. Takođe su procenjeni rezultati aktivnosti bolesti i radiografskog oštećenja zglobova. Statistički pristupi su uključivali korelacionu analizu, regresionu analizu i procenu ROC krive (recover operating characteristic).

**Rezultati:**

HALP indeks, ESR i RF bili su značajno povezani sa progresijom reumatoidnog artritisa. HALP indeks je pokazao jaku korelaciju sa aktivnošću bolesti (r=0,602, P&lt;0,001). ROC analiza je pokazala površinu ispod krive (AUC) od 0,858 za HALP; 0,841 za ESR i 0,924 za RF. Kada se kombinuju u multivarijantnom regresionom modelu, prediktivne performanse su se poboljšale (AUC = 0,943).

**Zaključak:**

HALP indeks, kao kompozitni biohemijski marker, u kombinaciji sa sedimentacijom eritrocita (SE) i reumatoidnim faktorom (RF) pruža značajne prognostičke informacije o progresiji reumatoidnog artritisa (RA). Ovi nalazi naglašavaju važnost laboratorijskih indeksa u usavršavanju prognostičkih procena i ističu njihovu potencijalnu ulogu u personalizovanom lečenju bolesti.

## Introduction

Rheumatoid arthritis (RA) is a chronic autoimmune disease that affects approximately 1% of the world's population, causing joint inflammation, bone erosion, and systemic complications [1][2]. It is characterized by symmetrical joint involvement, most commonly affecting the small joints of the hands, feet, and wrists [3][4][5]. RA can lead to significant disability, reduced quality of life, and increased risk of cardiovascular disease and other comorbidities [6][7]. The precise origin of RA remains incompletely understood, but it is believed to result from a sophisticated interaction among genetic, environmental, and immunological elements [8][9]. Despite advances in treatment, RA remains a challenging condition to manage, and early diagnosis and intervention are crucial for optimizing patient outcomes [10].

Despite advances in therapeutic interventions, the prediction of disease progression remains a complex and challenging task [11][12]. Current predictive models often rely on a combination of clinical, serological, and imaging markers to forecast the likelihood of progressive joint damage and functional decline in RA patients [13]. Commonly used prognostic markers include erythrocyte sedimentation rate (ESR) and rheumatoid factor (RF), which reflect systemic inflammation and the presence of autoantibodies, respectively [14][15].

Recently, researchers have shown increasing interest in utilizing multivariate regression models to integrate diverse biomarkers and improve the accuracy of prognostic predictions in RA [16]. The ESR and C-Reactive Protein (HALP index), calculated using routine complete blood count and serum albumin levels, has been suggested as an indicator of systemic inflammation and nutritional status in various diseases, including RA [17][18]. This index represents a composite measure that reflects the balance between inflammatory and nutritional markers, providing valuable insights into the overall health status of individuals [15][19]. In the context of RA, the HALP index may offer a more comprehensive assessment of disease activity and prognostic implications, potentially contributing to the refinement of predictive models and personalized management strategies for RA patients [5][20]. The combination of traditional markers such as ESR and RF with novel indices like the HALP index represents a promising approach to enhance the predictive power of existing clinical tools [21][22][23].

The aim of this study is to retrospectively investigate the predictive capability of a multivariate regression model that incorporates the Hybrid index of HALP index in combination with traditional markers such as ESR and RF. By evaluating the predictive performance of this novel model, we aspire to contribute to the growing body of knowledge aimed at refining prognostic approaches in RA, ultimately enhancing personalized therapeutic strategies and improving long-term outcomes for patients.

### Materials and methods

### Grouping criteria

The present study was a retrospective analysis that received approval from the Ethics Committee of Longyan First Affiliated Hospital of Fujian Medical University, following all relevant regulatory and ethical standards for retrospective research. For this retrospective investigation, informed consent was not required because only anonymized patient data were utilized, ensuring no risk of harm or influence on patient care. A total of 146 patients with RA admitted to our institution from December 1, 2022, to December 1, 2023, are included. Patients were categorized into a good prognosis group (n = 77) and a poor prognosis group (n = 69). The good prognosis group consisted of 28 males and 49 females, with an age range of 41 to 62 years and a mean age of 49.98 ± 5.64 years, while the poor prognosis group comprised 23 males and 46 females, whose ages spanned from 42 to 61 years, and had a mean age of 51.11 ± 6.22 years.

### Inclusion and exclusion criteria

Inclusion Criteria: Confirmed Diagnosis: Patients with a confirmed diagnosis of RA based on established diagnostic criteria [6]; Availability of Data: Patients for whom complete demographic, clinical, and radiographic data are available for analysis, including age, gender, disease activity scores, radiographic joint damage scores, HALP Index, ESR, and RF; Patients for whom prognostic data are available.

Exclusion Criteria: Comorbidities: Patients with significant comorbidities such as other autoimmune diseases (e.g., systemic lupus erythematosus) or chronic conditions (e.g., chronic kidney disease); Prior Treatment: Patients who have received specific immunomodulatory therapies, such as targeted biologic disease-modifying antirheumatic drugs (DMARDs) or Janus kinase (JAK) inhibitors [19][24]; Other Arthritic Conditions: Patients with overlapping arthritic conditions, such as psoriatic arthritis or ankylosing spondylitis.

### Laboratory data collection and biomarker assessment

Data collection focused on demographic information (age, gender, disease duration, smoking history, alcohol use, body mass index, living environment, hypertension, diabetes, and osteoporosis) together with a comprehensive panel of laboratory-derived biomarkers. Key biochemical and hematological parameters included the HALP index (calculated according to previously published definitions (20) as: HALP = [Hemoglobin (g/L) X Albumin (g/L) X Lymphocyte count (X10/L)] ± Platelet count (X10/L)), ESR, RF, C-reactive protein (CRP), anti-CCP antibodies, serum creatinine, and hemoglobin levels. Laboratory analyses were conducted in the hospital's central clinical laboratory using standardized automated platforms: hemoglobin, lymphocytes, and platelets were quantified on an XN-9000 automated hematology analyzer (Sysmex, Japan); albumin, CRP, and serum creatinine were measured with an AU5800 biochemical analyzer (Beckman, USA); ESR was determined with a MONITOR100 automated ESR analyzer; RF was assessed via nephelometry on a BN-II protein analyzer (Siemens, Germany); and anti-CCP antibodies were detected using a validated ELISA kit (Shanghai Kexin, China). Radiographic assessments (joint damage, bone erosion, joint space narrowing, and small joint erosions) were included as supportive outcome variables but the primary emphasis of this study was on laboratory biomarkers and their prognostic significance.

### Statistical analysis

Descriptive statistics were used to summarize the demographic and clinical characteristics of the study population. Subsequently, comparative analyses, including t-tests, chi-square tests, correlation analysis, regression analysis, and ROC analysis, were conducted to assess the relationships between the variables and the forecasting of disease progression among individuals with RA. Initially, multivariate logistic regression was performed to identify independent predictors of RA prognosis (good vs. poor). Subsequently, a K-nearest neighbor (KNN) regression model was applied to further assess the combined predictive performance of key laboratory indices (HALF, ESR, RF). The KNN model was chosen for its ability to capture potential nonlinear relationships among predictors and to improve the robustness of prediction in a relatively small sample without assuming normal data distribution. Model performance was evaluated using ROC curves and the AUC. Potential confounders, including age, sex, disease duration, and comorbidities, were assessed in preliminary univariate analyses, and variables with no significant between-group differences were included to minimize bias.

## Results

### Demographic characteristics

A total of 146 RA patients were included in the study, with 77 in the good prognosis group and 69 in the poor prognosis group (Table 1). The age of patients in the good prognosis group was 49.98 years (±5.64), while in the poor prognosis group, it was 51.11 years (±6.22). There was no significant statistical discrepancy observed between the two groups (t=1.14, *P*=0.256). The distribution of gender (M/F) was 28/49 in the good prognosis group and 23/46 in the poo^prognosis group, showing no substantial variation (χ^2^ =0.044, *P* =0.834). Similarly, no significant difference was observed in terms of disease duration, smoking history, alcohol consumption, BMI, living environment (coastal areas vs. inland areas), hypertension, diabetes, and osteoporosis between the two prognosis groups (all *P*>0.05). These results indicate that in patients with RA, the evaluated demographic characteristics show no significant differences between the good and poor prognosis groups, demonstrating comparability between the groups and laying the foundation for subsequent research.

**Table 1:** Demographic Characteristics of Rheumatoid Arthritis Patients.

Parameter	Good Prognosis Group (n=77)	Poor Prognosis Group (n = 69)	t/χ^2^	*P* Value
Age (years)	49.98±5.64	51.11±6.22	1.14	0.256
Gender (M/F)	28/49	23/46	0.044	0.834
Disease Duration (years)	4.74±1.85	5.30±2.52	1.512	0.133
Smoking History (n (%))	14 (18.18%)	17 (24.64%)	0.562	0.453
Alcohol Consumption (n (%))	27 (35.06%)	20 (28.99%)	0.369	0.543
BMI (kg/m^2^)	26.48±2.91	26.31±3.47	0.322	0.748
Living environment (n (%))			0	1
Coastal areas	69 (89.61%)	61 (88.6%)		
Inland areas	8 (10.39%)	8 (11.4%)		
Hypertension (n (%))	18 (23.38%)	16 (23.18%)	0	1
Diabetes (n (%))	14 (18.18%)	14 (20.29%)	0.013	0.91
Osteoporosis (n (%))	13 (16.88%)	12 (17.39%)	0	1

### HALP index and disease activity

Initially, this study conducted an evaluation of the HALP Index and disease activity scores in patients with RA, revealing significant statistical differences between the two groups (Table 2). The HALP Index was significantly lower in the good prognosis group (0.45±0.07) compared to the poor prognosis group (0.57±0.09) (t=8.909, *P* <0.001). The poor prognosis group displayed higher levels of ESR (0.44± 0.11 vs. 0.58±0.09, t=8.523, *P* <0.001), RF (172.38±16.54 vs. 128.54±15.25, t=12.053, *P *<0.001), C-reactive Protein (16.82 ±4.78 vs. 12.67±3.45, t=5.946, *P* <0.001), Anti-CCP Antibodies (196.48±30.22 vs. 158.25±25.67, t=8.189, *P *<0.001), Serum Creatinine (72.49± 10.61 vs. 91.94±13.26, t=9.73, *P* <0.001), and lower levels of Hemoglobin (11.67±1.61 vs. 12.88±1.57, t=4.599, *P* <0.001). These findings underscore the associations between the HALP Index, disease activity, and prognosis in RA patients, highlighting their potential as valuable indicators for prognostic assessment.

**Table 2:** Comparison of HALP Index and Disease Activity Scores between the two groups.

Parameter	Good Prognosis Group<br>(n=77)	Poor Prognosis Group<br>(n = 69)	t	*P* Value
HALP Index	0.45±0.07	0.57±0.09	8.909	P < 0.001
Erythrocyte Sedimentation Rate<br>(mm/hr) (mm /H)	0.44±0.11	0.58±0.09	8.523	P < 0.001
Rheumatoid Factor (IU/mL)	128.54±15.25	172.38±16.54	12.053	P < 0.001
C-reactive Protein (mg/L)	12.67±3.45	16.82±4.78	5.946	P < 0.001
Anti-CCP Antibodies (RU/mL)	158.25±25.67	196.48±30.22	8.189	P < 0.001
Serum Creatinine (μmol/L)	72.49±10.61	91.94±13.26	9.73	P < 0.001
Hemoglobin (g/L)	128.8±15.7	116.7±16.1	4.599	P < 0.001

### Radiographic joint damage scores

Subsequently, we compared the radiographic joint damage scores between the groups, and found statistically substantial variation in all parameters (Table 3). The Total Joint Damage Score was significantly lower in the good prognosis group (2.68 ± 1.55) compared to the poor prognosis group (5.34 ± 1.88) (t=9.254, *P* < 0.001). Likewise, the Progression Rate (12.81 ± 3.63 vs. 18.56 ± 4.74, t=8.17, *P* < 0.001), Bone Erosion Score (1.28 ± 0.82 vs. 2.59 ± 0.94, t=8.918, *P* < 0.001), and Joint Space Narrowing Score (1.97 ± 0.75 vs. 2.87 ± 0.66, t=7.669, *P* < 0.001) demonstrated significant distinctions when comparing the two groups. Furthermore, the distribution of radiographic damage showed no significant difference between the two groups (χ^2^ = 3.583, *P* = 0.167). However, erosions in small joints were significantly more prevalent in the poor prognosis group (57.97%) compared to the good prognosis group (32.47%) (χ^2^=8.578, *P* = 0.003). These results indicate a clear association between radiographic joint damage scores and the prognosis of RA, suggesting the potential of these indicators as prognostic markers for RA.

**Table 3:** Comparison of radiographic joint damage scores between the two groups.

Joint Damage Parameter	Good Prognosis Group<br>(n=77)	Poor Prognosis Group<br>(n = 69)	χ^2^/t	*P*-value
Total Joint Damage Score	2.68 ± 1.5	5.34 ± 1.88	9.254	*P* < 0.001
Progression Rate	12.81 ± 3.63	18.56 ± 4.74	8.17	*P* < 0.001
Bone Erosion Score	1.28 ± 0.82	2.59 ± 0.94	8.918	*P* < 0.001
Joint Space Narrowing Score	1.97 ± 0.75	2.87± 0.66	7.669	*P* < 0.001
Radiographic Damage Distribution			3.583	0.167
-Focal	13(16.88%)	18(26.07%)		
-Diffuse	11(14.29%)	14(20.29%)		
Erosions in Small Joints	25(32.47%)	40(57.97%)	8.578	0.003

### Correlation analysis

Correlation analysis between each index and RA revealed significant correlations for all parameters, with *P*-values less than 0.001 (Table 4), including HALP Index, ESR, RF, C-reactive Protein, Anti-CCP Antibodies, Serum Creatinine, Total Joint Damage Score, Progression Rate, Bone Erosion Score and Joint Space Narrowing Score. In contrast, a negative correlation was observed for Hemoglobin (*P* < 0.001). Additionally, Radiographic Damage Distribution showed a weak non-significant correlation (*P*=0.097), while Erosions in Small Joints displayed a significant, albeit relatively weaker correlation (*P*=0.002). These findings underscore the strong associations between the evaluated indices and the presence and progression of RA, emphasizing their potential utility in assessing disease activity and prognosis.

**Table 4:** Correlation analysis between each index and rheumatoid arthritis.

	r	R^2^	*P*
HALP Index	0.602	0.362	*P* < 0.001
Erythrocyte Sedimentation Rate	0.574	0.33	*P* < 0.001
Rheumatoid Factor	0.718	0.516	*P* < 0.001
C-reactive Protein	0.45	0.203	*P* < 0.001
Anti-CCP Antibodies	0.567	0.322	*P* < 0.001
Serum Creatinine	0.634	0.403	*P* < 0.001
Hemoglobin	-0.358	0.128	*P* < 0.001
Total Joint Damage Score	0.615	0.378	*P* < 0.001
Progression Rate	0.568	0.323	*P* < 0.001
Bone Erosion Score	0.599	0.359	*P* < 0.001
Joint Space Narrowing Score	0.536	0.287	*P* < 0.001
Radiographic Damage Distribution	0.138	0.019	0.097
Erosions in Small Joints	0.256	0.066	0.002

### Regression analysis

Multivariate logistic regression analysis revealed significant associations between each laboratory index and RA prognosis (Table 5). To further validate these findings and evaluate the combined predictive power of the HALP index, ESR, and RF, a KNN regression model was applied. The KNN approach, as a nonparametric learning algorithm, confirmed consistent trends with logistic regression and yielded strong discriminative ability. Regression analysis for each index and RA revealed significant associations for all parameters, with *P*-values less than 0.001, except for Radiographic Damage Distribution (*P*=0.098) and Erosions in Small Joints (*P *=0.002) (Table 5). The HALP Index (coef=18.722, Odds ratio=135114904.723), ESR (coef=0.224, Odds ratio = 1.251), RF (coef=0.101, Odds ratio=1.107), C-reactive Protein (coef=0.239, Odds ratio = 1.27), Anti-CCP Antibodies (coef=0.048, Odds ratio = 1.049), Serum Creatinine (coef=12.806, Odds ratio = 364264.723), Total Joint Damage Score (coef=0.904, Odds ratio=2.471), Progression Rate (coef=0.351, Odds ratio=1.42), Bone Erosion Score (coef=1.694, Odds ratio = 5.443), and Joint Space Narrowing Score (coef=1.817, Odds ratio = 6.154) displayed positive associations. Conversely, Hemoglobin showed a negative association (coef=0.479, Odds ratio = 0.619). These findings underscore the significant relationships between the assessed indices and the presence and progression of RA, further supporting their potential utility in evaluating disease activity and prognosis.

**Table 5:** Regression analysis between each index and rheumatoid arthritis.

	Coef	Odds ratio	B	Beta	P
HALP Index	18.722	135114904.723	6.007	18.722	P < 0.001
Erythrocyte Sedimentation Rate	0.224	1.251	5.843	0.224	P < 0.001
Rheumatoid Factor	0.101	1.107	6.075	0.101	P < 0.001
C-reactive Protein	0.239	1.27	4.943	0.239	P < 0.001
Anti-CCP Antibodies	0.048	1.049	5.828	0.048	P < 0.001
Serum Creatinine	12.806	364264.723	5.885	12.806	P < 0.001
Hemoglobin	0.479	0.619	4.096	-0.479	P < 0.001
Total Joint Damage Score	0.904	2.471	6.046	0.904	P < 0.001
Progression Rate	0.351	1.42	5.702	0.351	P < 0.001
Bone Erosion Score	1.694	5.443	5.932	1.694	P < 0.001
Joint Space Narrowing Score	1.817	6.154	5.543	1.817	P < 0.001
Radiographic Damage Distribution	0.363	1.437	1.654	0.363	0.098
Erosions in Small Joints	1.054	2.869	3.059	1.054	0.002

### ROC

The predictive value of various biomarkers and indices for disease progression in RA was evaluated through a ROC analysis (Table 6). The results demonstrate that the combined HALP index exhibited a high sensitivity of 88.4% and specificity of 72.7%, showing an AUC of 0.858 and Youden index of 0.611, indicating its potential as a predictive marker. Additionally, RF alone was found to have a sensitivity of 92.8% and specificity of 84.4%, with an AUC of 0.924 and Youden index of 0.772, suggesting its significant predictive capability. ESR also demonstrated reasonable predictive performance with a sensitivity of 78.3% and specificity of 77.9%, yielding an AUC of 0.841 and Youden index of 0.562. Conversely, CRP and Erosions in Small Joints displayed lower sensitivities and specificities, indicating less robust predictive capacity. Overall, these findings highlight the potential of the HALP index, ESR, and RF as valuable predictors of RA disease progression, supporting their use in the prediction of RA progression.

**Table 6:** Predictive Value of HALP Index, Erythrocyte Sedimentation Rate, Rheumatoid Factor Alone or in Combination for Disease Progression in Rheumatoid Arthritis.

	Sensitivities	Specificities	AUC	Ybuden index
HALP Index	0.884	0.727	0.858	0.611
Erythrocyte Sedimentation Rate	0.783	0.779	0.841	0.562
Rheumatoid Factor	0.928	0.844	0.924	0.772
C-reactive Protein	0.565	0.896	0.75	0.461
Anti-CCP Antibodies	0.783	0.74	0.826	0.523
Serum Creatinine	0.841	0.779	0.876	0.62
Hemoglobin	0.623	0.753	0.709	0.376
Total Joint Damage Score	0.928	0.636	0.86	0.564
Progression Rate	0.826	0.727	0.84	0.553
Bone Erosion Score	0.826	0.74	0.848	0.566
Joint Space Narrowing Score	0.87	0.61	0.808	0.48
Radiographic Damage Distribution	0.464	0.688	0.575	0.152
Erosions in Small Joints	0.58	0.675	0.628	0.255

**Figure 1:**
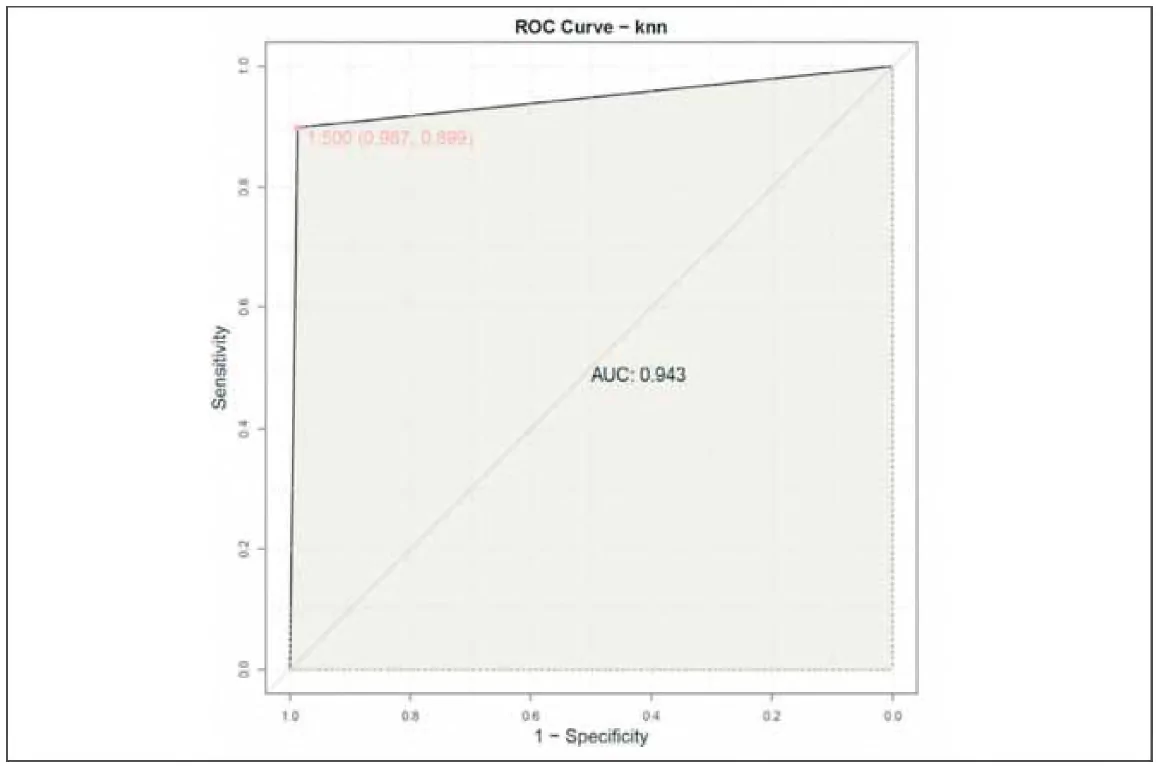
The predictive value of HALP index, ESR and RF in combination for the progression of rheumatoid arthritis.

Finally, this study utilized KNN multiple linear regression analysis to assess the predictive value of the HALP index, ESR, and RF combined for disease progression in RA, yielding an AUC value of 0.943 (Figure 1). These findings suggest that the combination of these three indicators holds significant predictive value for disease progression in RA, thereby serving as important predictive markers.

## Discussion

The findings of this retrospective study on the prediction of disease progression in RA patients provide valuable insights into the utility of combining classical laboratory markers with novel biochemical indices. Specifically, our multivariate regression model that integrates the HALP index with ESR and RF demonstrates significant prognostic potential. This approach reflects the growing trend in laboratory medicine of integrating routine hematological and biochemical parameters into comprehensive predictive tools for chronic disease management.

The demographic characteristics of the study cohort showed no significant differences between patients with good and poor prognoses, thereby ensuring comparability of the groups and strengthening the reliability of laboratory-based associations. Importantly, the HALP index was significantly linked to disease progression. Patients in the poor prognosis group presented with higher ESR, RF, C-reactive protein, anti-CCP antibodies, serum creatinine, and lower hemoglobin levels. These findings reinforce the HALP index as a valuable prognostic indicator, reflecting both systemic inflammation and nutritional status. This aligns with the work of Leetanaporn K. et al. [20], who demonstrated that the HALP index reflects the balance between inflammatory and nutritional markers, offering a more comprehensive biochemical perspective on disease status. Beyond HALF, other inflammation-based indices such as the neutrophil-to-lymphocyte ratio (NLR) and systemic immune-inflammation index (SII) have been widely studied in RA [25]. However, these parameters mainly reflect immune cell-based inflammation, whereas the HALP index simultaneously captures nutritional status (via hemoglobin and albumin) and immune response (via lymphocyte and platelet counts). Therefore, HALP may provide a more integrated perspective on disease activity and long-term prognosis.

In addition to serological and biochemical data, radiographic joint damage scores were significantly different between prognosis groups, supporting their role as complementary markers of disease progression. Nonetheless, it is noteworthy that biochemical indices, such as the HALP index and classical inflammatory markers, offer an earlier and more accessible means of prognostic evaluation, as they can be obtained through routine laboratory assays and automated analyzers widely available in clinical laboratories. In addition to classical inflammatory indicators such as ESR and CRP several composite biomarkers have been explored to assess disease activity in RA. Previous studies have demonstrated that elevated NLR, platelet-to-lymphocyte ratio, and SII levels correlate with higher disease activity and poorer functional outcomes in RA and other chronic inflammatory disorders [25][26][27]. However, these indices primarily reflect inflammatory cell dynamics and do not account for patients' nutritional or hematologic status. The HALP index, by combining hemoglobin and albumin (nutritional markers) with lymphocyte and platelet counts (immune-inflammatory markers), provides a more comprehensive evaluation of systemic inflammation and nutritional balance. This dual-domain approach may explain its stronger association with disease progression and radiographic damage observed in the present study, suggesting that HALP could complement or even outperform single-dimension inflammatory indices in prognostic assessment.

Correlation and regression analyses confirmed robust associations between these laboratory markers and RA progression, underscoring their utility in refining risk stratification. The ROC analysis further high-lighted the predictive value of the HALP index, ESR, and RF, particularly when used in combination. From a laboratory medicine standpoint, these results emphasize the importance of integrating multiple markers to achieve higher sensitivity and specificity, rather than relying on single indicators.

This study therefore contributes to the evolving framework of RA prognostic assessment by emphasizing the role of standardized biochemical and hematological parameters. The use of automated analyzers (hematology, biochemical, immunoassay platforms) ensures reproducibility and standardization of measurements, which is critical for the translation of these findings into routine laboratory practice. Our results align with the current trend in RA research described by Mukhtar M. et al., which emphasizes the development of comprehensive biomarker-based prognostic models to guide personalized therapeutic strategies and improve long-term outcomes [28].

The strengths of this work lie in its broad evaluation of serological, biochemical, and imaging markers, combined with multivariate statistical modeling, providing a holistic and laboratory-driven perspective on RA progression. However, certain limitations must be acknowledged. The retrospective design introduces inherent biases, and the single-center nature of the cohort may limit generalizability. Additionally, while biochemical indices such as HALP show promise, validation in larger, multicenter cohorts and the exploration of additional laboratory parameters (e.g., novel inflammatory biomarkers, proteomic or metabolomic signatures) will be essential to strengthen their clinical applicability.

In conclusion, this study highlights the prognostic value of routine laboratory indices, particularly the HALP index in combination with ESR and RF, in predicting RA progression. These findings underscore the pivotal role of laboratory medicine in delivering accessible, reproducible, and clinically meaningful biomarkers for personalized management of RA patients.

## Conclusion

This study demonstrates that the HALP index, in combination with ESR and RF, provides strong prognostic value for predicting disease progression in RA. Significant correlations between these laboratory markers and disease activity, radiographic joint damage, and prognosis highlight their utility as accessible and reproducible indicators. From a laboratory medicine perspective, the HALP index integrates hematological and biochemical parameters, reflecting both systemic inflammation and nutritional status. Its combined use with ESR and RF enhances predictive accuracy, as shown by regression and ROC analyses. This underscores the importance of incorporating standardized laboratory indices into multivariate models to improve prognostic assessments and guide individualized management strategies. Overall, these findings emphasize the pivotal role of clinical biochemistry in RA prognosis. Validation in larger, multicenter cohorts and exploration of additional biomarkers are needed to confirm and extend these results, ultimately supporting the broader application of laboratory-based tools in personalized care for RA patients.

## Dodatak

### Acknowledgements

The authors would like to express their gratitude to all the participants and staff who were involved in this study.

### Author contributions

XX. X.: Conceptualization, Investigation, Methodology, Data curation, Formal analysis, Writing - original draft; XH. H., YF L.: Investigation, Methodology, Data curation, Formal analysis, Software; DF Z.: Conceptualization, Project administration, Supervision, Writing - review and editing.

### Data availability statement

The data involved in the present study can be provided under reasonable request.

### Funding statement

This research did not receive any specific grant from funding agencies in the public, commercial, or not-for-profit sectors.

### Ethics approval statement

This study received approval from the Ethics Committee of Longyan First Affiliated Hospital of Fujian Medical University, following all relevant regulatory and ethical standards for retrospective research.

### Patient consent statement

For this retrospective investigation, informed consent was not required because only anonymized patient data were utilized, ensuring no risk of harm or influence on patient care.

### Permission to reproduce material from other sources

Not applicable.

### Clinical trial registration

Not applicable.

### Conflict of interest statement

All the authors declare that they have no conflict of interest in this work.
